# COVID-19 Induced Taste Dysfunction and Recovery: Association with Smell Dysfunction and Oral Health Behaviour

**DOI:** 10.3390/medicina58060715

**Published:** 2022-05-26

**Authors:** Georgia Catton, Alexander Gardner

**Affiliations:** Department of Restorative Dentistry, Dundee Dental Hospital and School, University of Dundee, Dundee DD1 4HR, UK; g.s.catton@dundee.ac.uk

**Keywords:** COVID-19, taste dysfunction, oral health, dental care

## Abstract

*Background and Objectives*: Disruption to taste and smell are common symptoms of COVID-19 infection. The current literature overlooks taste symptoms and tends to focus on the sense of smell. Persisting cases (>28 days) of taste dysfunction are increasingly recognised as a major future healthcare challenge. This study focuses on the severity and recovery of COVID-19 induced taste loss and association with olfactory symptoms, lifestyle and oral health factors. *Materials and Methods*: This study was a cross-sectional survey comparing 182 rapid taste recovery participants (≤28 days) with 47 participants with prolonged taste recovery >28 days. Analyses of taste loss in association with smell loss, age, sex, illness severity, diet, BMI, vitamin-D supplementation, antidepressants, alcohol use, smoking, brushing frequency, flossing, missing teeth, appliances and number of dental restorations were conducted. Differences in the severity of the loss of sour, sweet, salt, bitter and umami tastes were explored. *Results*: Both the severity and the duration of taste and smell loss were closely correlated (*p* < 0.001). Salt taste was significantly less affected than all other taste qualities (*p* < 0.001). Persisting taste loss was associated with older age (mean ± 95% CI = 31.73 ± 1.23 years vs. 36.66 ± 3.59 years, *p* < 0.001) and reduced likelihood of using floss (odds ratio ± 95% CI = 2.22 (1.15–4.25), *p* = 0.047). *Conclusions*: Smell and taste loss in COVID-19 are closely related, although a minority of individuals can experience taste or smell dysfunction in the absence of the other. The taste of salt may be less severely affected than other taste qualities and future work exploring this finding objectively is indicated. The association of flossing with rapid taste recovery adds to the growing evidence of a link between good periodontal health and favourable COVID-19 outcomes.

## 1. Introduction

Alteration or loss of sense of smell (olfaction) and taste (gustation) were noted as critical symptoms early in the SARS-CoV-2 pandemic and were the most definitive symptoms of infection [1,2]. It has been shown that sensory symptoms can perform comparably with a polymerase chain reaction (PCR) test of nasopharyngeal swabs. Smell loss has been shown to demonstrate a sensitivity of 65% and specificity of 97%, whereas the corresponding values for a PCR swab are 87% and 97% [3,4]. The high specificity in particular means sensory symptoms are strongly indicative of the disease and public health guidance quickly reflected this, encouraging isolation in the advent of rapid onset smell or taste disturbance [5]. Machine learning models have demonstrated that sensory symptoms in conjunction with machine learning can yield sensitivity of 82%, with smell loss being more characteristic of a true positive than taste loss [6]. Estimates of the prevalence of sensory symptoms vary and range from 87% for smell loss and 56% for taste loss, down to under 15% for either symptom [7,8]. It has been noted that the more recent Omicron strain of the virus, which emerged in Winter 2021, appears to result in less instances of sensory disturbance compared with the predominant strains earlier in the pandemic. Data supports a decrease in the prevalence of olfactory symptoms from 63% to 25% and a decrease in the prevalence of gustatory symptoms from 57% to 27% [9]. Despite a significant decrease in prevalence, sensory symptoms are still seen to affect considerable proportions of infected individuals.

In health, the sense of taste is perceived when taste molecules or ions bind to G-protein coupled receptors (GPCR) expressed within taste receptor cells, which are housed in clusters known as taste buds. Taste buds are primarily located within specialised papillae on the tongue. These include fungiform papillae anteriorly and foliate and circumvallate papillae posteriorly [10]. Upon binding to a GPCR, a signalling cascade mediated by cyclic adenosine monophosphate (cAMP) or inositol triphosphate results in neurotransmitter release at a basal synapse, ultimately triggering an action potential. The nerves that carry taste to the brain in humans are facial, glossopharyngeal and vagus nerves (7th, 9th and 10th cranial nerves, respectively) [11,12]. Smell is detected by an analogous receptor–molecule binding mechanism. Specialised polarised olfactory receptor cells are housed within the olfactory epithelium at the top of the nasopharynx. Odorant molecules are present within inhaled air and bind to GPCRs, initiating a cAMP mediated action potential. This is relayed via the olfactory bulb to the brain by the olfactory nerve, the first cranial nerve [13].

A key difference between taste and smell in humans is the number of tastes compared with the number of smells that can be detected. Taste is currently understood to comprise five primaries, or basic, tastes. These include sweet, bitter, umami, salt and sour. Salt and sour tastes are thought to be ionic, caused by sodium or hydrogen ions, respectively. Sweet taste is caused by molecules such as sucrose or artificial sweeteners such as aspartame. Bitter tastes are caused by a wide range of molecules including many plant alkaloids. Umami taste is a savoury taste caused primarily by monosodium glutamate (MSG) or nucleotides such as inosine or guanosine monophosphate [14]. Humans have up to 400 different odour receptors, which when activated in different combinations allows potential for recognising up to one trillion different smells [15,16]. Due to this vast array of odours, attempts to cluster different smell molecules based on similar odour qualities have been made using ten different categories [17]. 

It remains unclear how COVID-19 damages the senses of smell and taste. A leading theory is that the virus can bind to, enter and destroy chemosensory receptor cells or neurones via angiotensin converting enzyme 2 (ACE-2) that is expressed by these cells [18]. An alternative mechanism may be via indirect damage to olfactory and gustatory tissue by increased inflammatory molecules such as interleukin-6 (IL-6), tumour necrosis factor alpha (TNF-α) and gamma interferon (γ-INF). These inflammatory markers may impede cell function, including interfering with their natural turnover [19]. Other mechanisms include hypoxic damage interfering with the central perception of taste and smell and changes in oral environment via salivary gland damage affecting function of taste receptor cells [20]. 

**Figure 1:**
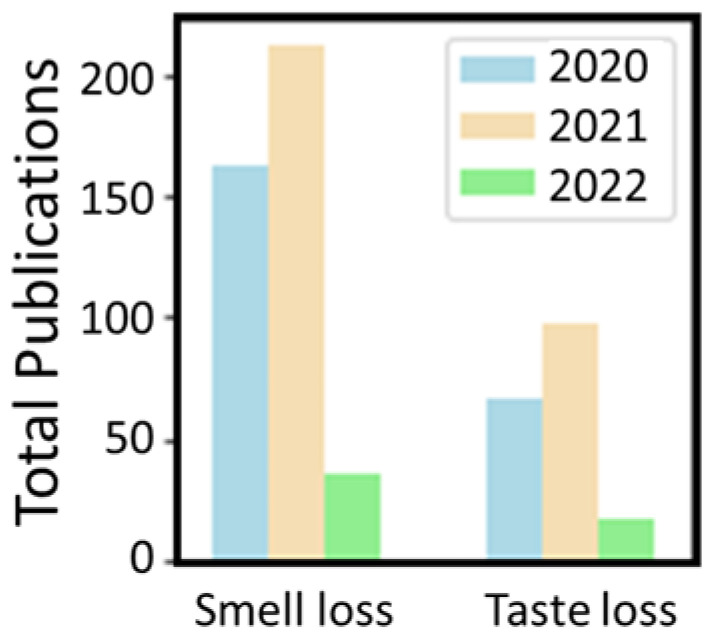
Comparison of total publications on either smell or taste loss and COVID-19 infection since the pandemic began. Data generated by searching the Web of Science using the terms “COVID taste OR COVID gustatory NOT smell NOT olfactory” and “COVID smell OR COVID olfactory NOT taste NOT gustatory” in the title field.

A general overview of the literature on taste and smell loss following COVID-19 infection reveals that studies of smell loss seem to be more than twice as prevalent as studies of taste loss. This is evidenced in Figure 1, which illustrates the number of publications identified by literature searches for the respective symptoms associated with COVID-19 since the start of the pandemic. At the time of searching (April 2022), 410 articles on smell loss and COVID-19 and 178 on taste loss and COVID-19 were returned.

The reason for the discrepancy between smell and taste loss may be rooted in several reasons. Firstly, true loss or change in taste was rare before COVID-19, and in many cases is secondary to the disruption of olfaction as much of what is experienced during eating and drinking is attributable to the sense of smell. Recent meta-analysis has added weight to the argument of a genuine disruption to the sense of taste in COVID-19 infection [21]. Another reason may be that due to the lower prevalence of taste disturbance in COVID-19, and thus the lower predictive value of taste symptoms, there has been a tendency in researchers to focus on olfactory symptoms and overlook gustatory symptoms. Another overlooked factor in the study of COVID-19 taste loss is the effect on individual taste qualities which include sweet, bitter, sour, salty and umami. Efforts to capture this information have been recommended in future studies [22].

Thanks to global public health efforts, including vaccination and population level screening, SARS-CoV-2 is moving from a pandemic to an endemic status in many countries. The challenges posed by the virus are not yet over, however, with a major concern being the management of morbidity in survivors of the infection. Many studies find that the majority of individuals recover chemosensory function rapidly, with upwards of 80% returning to normal within two months [23,24,25], and in some studies recovery is even faster [8,26]. Worryingly, there are increasing reports of delayed sensory recovery with chemosensory effects being reported to persist for over a year in up to 30% of cases [27]. Furthermore, there are reports of taste disruption persisting in around one third of cases at six months, whereas smell disruption persisted in only 15% of cases at the same time point [28]. Other work reports that olfactory symptoms show a slower recovery than taste disturbance [29]. Persistent symptoms of COVID-19 have been reported from the early stages of the pandemic, sometimes referred to as “long COVID”, although there is a lack of clarity over definitions [30]. A four-week cut off for the persistence of symptoms has been adopted within the definitions of the Centers for Disease Control (CDC) in the United States and the National Health Service (NHS) in the United Kingdom. It is recognised that persisting sensory disruption following COVID-19 has a significant quality of life impact on patients, with a lack of data about the management of the conditions of concern to both patients and clinicians [31,32]. 

The aim of the present study was to evaluate the loss and recovery of taste function in COVID-19. This included a granular analysis of the intensity and duration of taste loss in relation to smell loss, a comparison of intra-individual evaluation of the intensity of loss of individual taste qualities and an analysis of lifestyle, behavioural and oral health related factors that may be associated with prolonged (>28 day) taste loss.

## 2. Materials and Methods

### 2.1. Ethical Approval

This study was approved by the University of Dundee School of Health Sciences and Dentistry ethics committee (ref: UOD\SDEN\STAFF\2020\017, 25 June 2020).

### 2.2. Survey Design and Dissemination, Data Gathering and Processing

A full description of methodology has been previously published [25]. In brief, a survey was designed using JISC Online Surveys (JISC, Bristol, UK). Following written approval from website administration, the survey was hosted from March to August 2020 at https://www.reddit.com/r/COVID19positive (accessed on 10 March 2021). The survey was open to individuals >18 years old who had suffered COVID-19 infection diagnosed by PCR test, a physician or self-diagnosis, where symptoms included acute changes to smell and/or taste. The full survey can be found in Supplementary Figure S1 (information for participants and questionnaire).

Target variables were maximum severity of smell loss and taste loss and duration of smell and taste recovery to baseline levels. The severity of individual taste modalities (sweet, sour, bitter, salty and umami) were also asked. Umami taste was described as that of savoury food or food containing monosodium glutamate (MSG). The duration of taste loss and smell loss was defined by the time taken to return to normal baseline as subjectively assessed by the individual. Thus, partial recovery of some taste or smell was still considered an incomplete recovery. The duration of sensory loss was defined by continuous variables along with age, BMI and daily fruit and vegetable intake. Severity of taste and smell loss were ordinal variables, alongside severity of overall illness and severity of congestion, which were defined on an 11-point (0–10) scale, defined as 0 = no effect and 10 = total loss of sensation. Further ordinal variables were brushing frequency (1,2 or >2 times daily), alcohol consumption frequency (monthly, weekly, daily, never), smoking (never, former, current) and number of dental restorations (0, 1–5, 5–10, >10). Binary variables were biological sex, vitamin-D supplementation, antidepressant use, use of floss/interproximal cleaning, missing teeth (not lost due to trauma, orthodontics or impaction) and appliance wearing. Statistical analysis of ethnicity was precluded by low numbers of African and South Asian respondents.

### 2.3. Sample Size and Statistical Power

Based on a 95% confidence level, *p* = 0.05 and a precision of ±5%, a sample size of 400 was deemed necessary as a representation of the study population [33]. Preliminary data exploration suggested a cut off of ~30 days would yield a sampling ratio of approximately 4:1 (recovery ≤28 days: recovery >28 days). This distribution ratio was sufficient to detect effect sizes differing by 15–20% between groups with alpha = 0.05 and power = 0.8, based on a sample distribution of 229 participants (182 rapid recovery: 47 prolonged recovery).

### 2.4. Statistical Analyses

Python3 (Centrum Wiskunde & Informatica, Amsterdam, The Netherlands) and SPSS 27 (IBM, Armonk, NY, USA) were used to analyse and visualise data. Normality tests (QQ plots and Shapiro–Wilk test) were conducted on continuous data. The relationships between intensity of taste loss and intensity of smell loss and congestion severity were explored by Spearman’s rank correlation. The timing of the recovery of smell and taste function was visualised. Differences between the degree of taste loss for the different taste sensations were analysed by Friedman test with Bonferroni-adjusted pairwise comparisons. Differences in the recovery time for smell and taste loss were explored. Differences between rapid recovery of taste (≤28 days) and prolonged recovery (>28 days) were then explored for the remaining variables. Means were compared by two-tailed *t*-test or Mann–Whitney test where appropriate. Binary variables were compared using Fisher’s exact test and categorical variables were compared by Pearson’s chi-squared test and odds ratios calculated.

## 3. Results

### 3.1. Relationship between Resolution of Smell and Taste Loss

**Figure 2:**
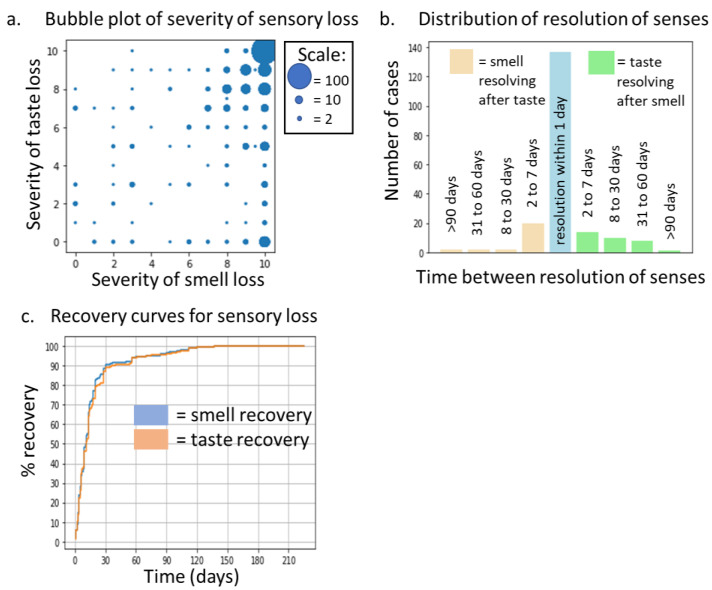
(**a**) Bubble plot showing the relationship between taste and smell loss severity, *n* = 421. (**b**) Plot indicating the differential time for resolution of taste and smell loss, *n* = 207. (**c**) Recovery curve for both taste and smell loss, *n* = 207.

A total of 421 participants reported some degree of smell or taste disruption. The severity of these symptoms was highly significantly correlated, ρ = 0.47, *p* < 0.0001. Data are displayed in a bubble plot in Figure 2a. Both senses were most commonly reported as completely lost (rating of 10), although a tendency for smell loss to be rated as more severe than taste loss as evidenced when comparing the distribution of responses in the bottom right to the top left. Of these participants, 207 reported full recovery of both senses at the time of completing the survey. Duration of sensory recovery was also significantly correlated, ρ = 0.83, *p* < 0.0001. Figure 2b indicates that in 70% of cases individuals recovered both senses within a day of each other, although there were cases of either taste or smell recovering first by up to 90 days in a small number of cases. Recovery curves (Figure 2c) indicate that taste recovery was very similar to that of smell recovery. Within 14 days, 64% of cases had resolved and within 30 days, 87% had resolved, rising to 96% resolution within 90 days and only two cases (<1%) persisted beyond 120 days. No association was observed between congestion severity and taste loss intensity (ρ = 0.04, *p* = 0.37).

### 3.2. Differences between Severity of Loss of Different Taste Qualities

**Figure 3:**
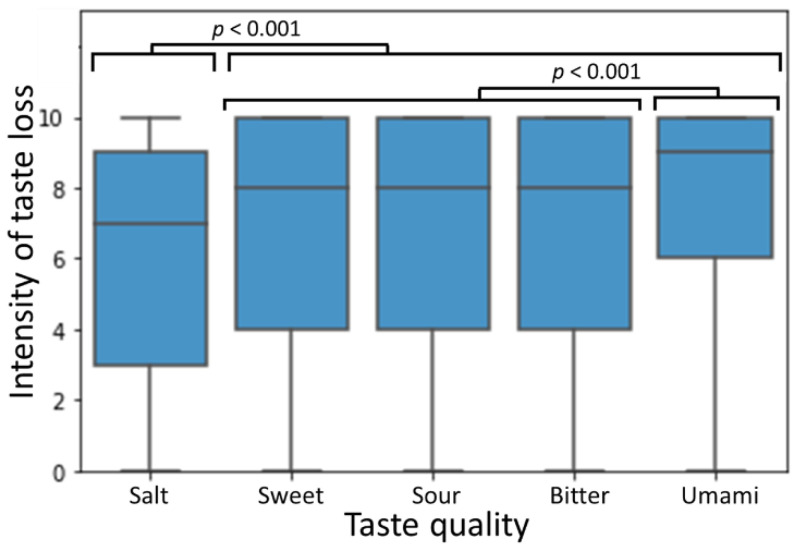
Comparison of intensity of taste sensation loss for the five primary taste modalities. *p*-values are for Friedman test with Bonferroni-adjusted post hoc pairwise comparisons between all groups, *n* = 384.

The differences in the severity rating of each different taste quality were analysed for all individuals reporting some degree of taste loss. For all tastes the degree of effect ranged from 0 (no change) to 10 (complete inability to detect the taste). Data are shown in Figure 3, comparing individuals’ ratings of each taste quality by Friedman test with Bonferroni-adjusted post hoc pairwise comparisons. Salt taste was significantly less affected than all other tastes (*p* < 0.001) and umami taste was significantly more severely affected than salty, sweet or sour tastes, which were all affected to a comparable degree. 

### 3.3. Differences between Rapid (≤28 Days) and Prolonged (>28 Days) Taste Recovery

At the time of completing the survey, 182 individuals reported a resolution of taste loss in ≤28 days and 47 individuals reported taste loss persisting >28 days. A full breakdown of the statistical analyses is presented in Table 1. Statistically significant differences between groups were found for age and the use of floss. Individuals with prolonged taste recovery were on average older than the rapid recovery group. Mean and 95% CI age for rapid taste recovery was 31.73 (30.50–32.96) years compared with 36.66 (33.07–40.25) for prolonged recovery, *p* = 0.001. A higher proportion of flossers was found in the rapid recovery groups compared with the prolonged recovery group. Distributions were 136/182 (75%) in the rapid taste recovery compared with 28/47 (60%) in the prolonged taste recovery, odds ratio (95% CI range) = 2.22 (1.15–4.25), *p* = 0.047. None of the other lifestyle and oral health parameters analysed differed significantly. A result approaching significance was found for illness severity when comparing rapid taste loss recovery, 4.42 (4.15–4.70) vs. prolonged taste loss recovery, 4.98 (4.42–5.54), *p* = 0.061. 

## 4. Discussion

### 4.1. Relationship between Taste and Smell Loss

Whilst taste loss in COVID-19 infection is accepted to be closely linked to olfactory symptoms, few studies quantify the strength of the relationship. The present work finds a moderate yet highly significant correlation (ρ = 0.47, *p* < 0.0001) between the intensity of taste and smell loss. One study asked participants to rate their degree of taste and smell dysfunction from 0 to 10 using a Visual Analogue Scale (a similar approach to the present work) report a Pearson’s correlation of r = 0.91 (*p* < 0.0001, *n* = 162). This reflects a stronger degree of relationship than is implied from our data, however fewer participants were used in that study [34]. Other findings suggest the correlation may be closer to the value reported in the present study (Pearson’s r = 0.53), however the methodology used to assess taste and smell is not clear [35]. Further work in a larger sample confirms a weak but significant correlation between self-assessed smell and taste loss (ρ = 0.25, *p* < 0.01, *n* = 1172) [26]. 

Our results confirm that the correlation between the recovery time for smell and taste was stronger than the correlation between the severity of smell and taste loss (ρ = 0.83 vs. ρ = 0.47). Cecchetto et al., report that the recovery of smell and taste were both strongly associated with each other in multivariate analyses of sensory recovery, with a correlation coefficient between 0.86 and 0.91 [36]. Lee et al., imply a very close pattern of recovery between both senses, although it is not quantified [8]. Our observation that smell loss in the absence of taste loss appears more prevalent than the opposite is also supported by Lee et al., who observe this pattern of loss in 36% more cases than taste loss in the absence of smell loss. Our finding that smell and taste tend to resolve simultaneously (80% within one day of each other), with a small number of cases showing resolution of smell or taste first, with roughly equal prevalence, has not been reported elsewhere to our knowledge. We did not observe any significant relationship between congestion severity and taste loss severity. No link between congestion intensity and taste loss has been explored in the literature, although there are mixed reports of a link between the duration of smell loss and the presence of congestion in COVID-19 [23,37]. A significant link between symptom intensity has not been found [38]. The knowledge of the resolution time for COVID-19 is of key importance in making evidence-based guidelines for healthcare access in the post-pandemic world. International guidelines support deferring dental care in those with COVID-19 symptoms due to the risk of aerosol transmission [39]. Given that sensory symptoms can persist for weeks or months after an individual ceases to be able to transmit the infection, it is clearly not practical for dental care to be delayed this long. 

### 4.2. Differences between Individual Taste Modalities

The majority of the literature on taste loss in COVID-19 treats taste as a single variable, rather than exploring the individual taste qualities [21]. Our study suggests that salt taste was less severely affected than sweet, sour, bitter and umami tastes and that umami taste was more severely affected than sour, sweet and bitter tastes. A recent objective study of taste thresholds in COVID-19 reported a decrease in salt threshold (i.e., greater ability to detect salt) in COVID-19 patients compared with regular controls. Sour, sweet and bitter taste thresholds all increased, and umami was not studied [40]. Other work finds that bitter and sour taste may be less affected than salt and sweet taste when participants rated intensity of these substances on a scale out of 100, however an appropriate control was absent [41]. Contrastingly, objective changes in sweet and salt taste have not been confirmed in separate works, with authors suggesting the taste losses are related to reduced retronasal olfaction in relation to smell loss [42,43]. 

### 4.3. Differences between Prolonged and Rapid Taste Recovery

Given the close relationship between taste and smell recovery that we observe, it is unsurprising that the associated differences between prolonged (>28 day) and rapid (≤28 day) taste recovery are in line with our previous work on smell recovery [25]. The main difference was that illness severity was not significantly associated with recovery, although the relationship approached significance with *p* = 0.06. Age was positively associated with prolonged recovery of taste. Whilst some reports suggest age may be associated with the recovery of smell loss [37], systematic reviews suggest the majority of studies do not find an association between age and sensory recovery [44]. Regarding the association of flossing with taste recovery, it has been reported that poor periodontal health is associated with increased inflammation and poorer COVID-19 outcomes, although sensory symptoms were not specifically assessed [45,46]. Some of the oral health factors assessed, including brushing frequency and wearing of appliances, have been associated with taste sensitivity in healthy individuals [47,48]. These may arise from alterations in the oral environment and the biofilms that coat the tongue and the taste receptors [49]. While we observed a significant association with flossing, there is evidence that interdental brushing may be slightly better for periodontal health than flossing [50], thus this would represent an interesting avenue for future study. Tongue brushing, which has been shown to improve taste perception in the absence of disease, represents a further key variable that requires future study [51,52]. A targeted approach to recruitment would be indicated as it appears to be a relatively uncommon practice, with only 18% of people engaging in tongue brushing daily, which is necessary for optimal benefit [53,54]. 

Of the variables with no relationship with taste recovery, few have been explored previously. Smokers, whilst known to have diminished taste in health, have been observed to recover taste loss in COVID-19 infection faster than non-smokers [36]. The reason for this is not clear but may be related to short-term abstaining from smoking during illness causing a net increase in taste sensitivity. Alcohol consumption, antidepressant use and diet have not been directly studied in association with taste loss. Vitamin D levels have been found to be significantly different in those with or without taste loss in COVID-19 [55]. Regarding BMI, the duration of taste loss did not significantly differ between overweight/obese and normal weight participants, although those with higher BMI did appear to have a greater frequency of taste loss but not smell loss [56]. Sex is perhaps the most widely studied variable in associated with sensory disruption. A systematic review finds only four of seventeen articles found an association between sex and sensory symptoms [44]. Other studies find no relation between sex and recovery time for taste [23], although one study found an association (longer recovery in females) in a sub-group of their participants (Italian vs. British) [57].

Mechanistically, the loss of taste and smell in COVID-19 infection is still unclear. The role of ACE-2, which is expressed on the tongue and the taste receptors, is recognised in facilitating viral entry and is believed to have a pivotal role in the aetiology [18]. Recent work confirms that viral particles are detectable in the taste receptors of 75% of COVID-19 positive cadavers [58]. It has also been demonstrated in vitro that direct ACE-2 mediated viral entry into olfactory and gustatory neurones [59]. Salivary glands may present a further mechanism of viral entry and dissemination [60]. Collectively, these studies may imply there are multiple mechanisms by which SARS-CoV-2 can affect taste and may explain why the present study finds that some individuals suffer only loss of taste or smell independent of the other. The finding of flossing being associated with rapid taste recovery may also reflect a mechanistic link with viral entry. Flossing is linked to reduced plaque and bleeding, a clinical indicator of inflammation, as well as reduced bacteraemia [50,61,62]. Virion entry via inflamed periodontal pockets is a further source of COVID-19 infection [63], and raised inflammatory markers such as Interleukin-6 and C-reactive protein are implicated in sensory disruption in COVID-19 infection as well as gum disease [64,65]. Thus, flossing may reduce viral entry and dissemination in hosts as well as create a less inflammatory local environment that would predispose to sensory symptoms. Indeed, there is evidence from meta-analysis that flossing in combination with brushing confers gingival health benefits over brushing alone [50]. This is an effect that grows stronger from adopting the habit, up to a six-month follow-up. While infrequent brushing is associated with poor periodontal health, all participants in the present analysis brushed at least once a day, which is sufficient frequency not to affect periodontal health significantly [66,67].

### 4.4. Limitations and Future Work

The present work contains several limitations. Firstly, as it was conducted during the first wave of global lockdowns, mass testing was not readily available and thus participants with symptomatic diagnoses without PCR confirmation were included. Given the roughly equivalent specificity of sensory symptoms to PCR test and our analyses of individuals displaying sensory symptoms, this did not have much bearing on results when comparing PCR positive participants only [25]. A major challenge in the study of taste disorders in COVID-19 is the reliance of self-reported data rather than objective tests. Literature reviews suggest that the proportion of studies using self-assessed data is only about 8% [21,68]. The literature suggests that a reasonable correlation between self-reported and objectively tested smell function exists [34,69], however this is less clear for taste dysfunction in COVID-19. There is evidence that many individuals self-reporting taste dysfunction in COVID-19 display normal taste function when objectively tested [42], although subjective smell and taste scores do correlate significantly [42,70]. The present study made comparisons of self-assessed taste scores within individuals (i.e., paired comparisons), hence inter-individual variance in self-assessment should be negated by this. When considering between group differences, the primary outcome of taste recovery time is a more objective metric as individuals again report their sensation relative to their own baseline. Nevertheless, there is a clear need for future studies to undertake objective psychophysical assessments of taste and smell. As globally we are seeing increased cases of persisting sensory dysfunction in individuals who are no longer acutely infectious, these studies will become increasingly feasible [21]. Further challenges to the present work include the relatively uneven recovery, as the ratio of recovery before and after 28 days was about 3.9:1; therefore, it is possible that we may lack sufficient power to detect genuine differences between the groups with smaller effect sizes. This must be considered alongside the likely clinical and biological significance however, as this would be expected to be small in these circumstances. Finally, we must caution the lack of any causative associations that can be drawn from an observational study as this. For example, flossing cannot be claimed to be protective of long taste recovery in COVID-19 and may simply reflect an individual with greater health awareness who is engaging in other protective behaviours not assessed in this study. The *p*-value was only just below the 0.05 threshold and would not be significant following adjustment for multiple testing. As this work is primarily exploratory to identify potential relationships for further exploration, a high false discovery rate would be tolerable to mitigate the risk of Type I errors. Future prospective studies of a greater number, ideally clinically measuring periodontal pockets and bleeding, will aid confidence in this observed result. The observations of individuals with either only taste or smell loss in the absence of the other merits further investigation. A selective comparison between individuals experiencing only taste dysfunction versus only smell dysfunction would contribute to the mechanistic understanding of sensory dysfunction in COVID-19. 

## 5. Conclusions

The present study suggests that taste dysfunction is closely correlated to smell dysfunction in COVID-19, both for intensity and duration of symptoms. The relationship was stronger for duration (ρ = 0.83) than intensity (ρ = 0.47), although both relationships were highly significant (*p* < 0.0001). There was a tendency for smell loss to occur more commonly in the absence of taste loss than taste loss to occur in the absence of smell loss. This knowledge can help target individuals for future study that may aid the understanding of the mechanistic effects of sensory loss in COVID-19. Salt taste was less affected than sweet, sour, bitter and umami taste and umami taste was more severely affected than bitter, sour and sweet taste. Future work involving the objective measurement of taste thresholds is indicated, as the current literature is heavily biased towards self-reported data. This will become more feasible in the future as taste disturbance can persist long after acute infection in some individuals. Persisting taste dysfunction >28 days was significantly associated with older age and reduced use of floss. Non-significant factors included sex, diet, BMI, vitamin-D, antidepressants, alcohol use, smoking, brushing frequency, missing teeth, appliances and number of restorations. Flossing may be an important marker of local inflammation and gingival pocketing, which may aid in viral entry and creation of a local environment triggering neurological dysfunction. Future study including clinical measurement of periodontal health is indicated to aid in the understanding of sensory dysfunction in COVID-19. The present results add to the growing literature that taste and smell dysfunction can persist for >28 days in approximately one quarter of COVID-19 infections. We would advise that guidelines created to advise the public and healthcare professionals about COVID-19 symptoms consider that taste and smell loss may persist beyond the transmissible stage of infection and thus should not automatically be a barrier to seeing a medical or dental professional. 

## Figures and Tables

**Table 1:** Comparison of short (≤28 days) and long (>28 days) taste loss. For Fisher’s exact test and Chi^2^ test observed values are shown followed by expected values in brackets. *—significant at *p* < 0.05, **—significant at *p* < 0.01.

Variable	Test	Short Smell Loss (≤28 Days) Mean ±95% CI		Long Smell Loss (>28 Days) Mean ±95% CI		Test Statistic	*p*-Value
Age	*t*-test	31.73 (30.50–32.96)		36.66 (33.07–40.25)		−3.23	0.001 **
BMI	*t*-test	26.74 (25.88–27.60)		26.16 (24.70–27.62)		0.62	0.54
Fruit/vegetable intake	*t*-test	2.98 (2.75–3.22)		3.20 (2.65–3.75)		−0.79	0.43
Illness severity	Mann–Whitney	4.42 (4.15–4.70)		4.98 (4.42–5.54)		−1.87	0.061
Smell loss severity	Mann–Whitney	8.46 (8.07–8.84)		8.36 (7.53–9.18)		−0.31	0.75
Taste loss severity	Mann–Whitney	7.79 (7.41–8.12)		7.56 (6.82–8.31)		−0.69	0.49
Sex	Fisher’s exact	M	70 (65)	M	12 (17)	NA	0.13
		F	112 (117)	F	35 (30)		
Vitamin D	Fisher’s exact	No	154 (157)	No	44 (41)	NA	0.15
		Yes	28 (25)	Yes	3 (6)		
Antidepressant	Fisher’s exact	No	161 (161)	No	42 (42)	NA	1.00
		Yes	21 (21)	Yes	5 (5)		
Flossing	Fisher’s exact	No	46 (52)	No	19 (13)	NA	0.047 *
		Yes	136 (130)	Yes	28 (34)		
Missing teeth	Fisher’s exact	No	153 (153)	No	39 (39)	NA	0.81
		Yes	25 (25)	Yes	7 (7)		
Appliances	Fisher’s exact	No	147 (147)	No	38 (38)	NA	1.00
		Yes	35 (35)	Yes	9 (9)		
Brushing freq.	Pearson’s Chi^2^	Daily	58 (53)	Daily	9 (14)	4.25	0.12
		2 × day	114 (116)	2 × day	32 (30)		
		>2 × day	9 (11)	>2 × day	5 (3)		
Alcohol freq.	Pearson’s Chi^2^	Never	35 (37)	Never	11 (9)	1.34	0.72
		Monthly	43 (43)	Monthly	11 (11)		
		Weekly	64 (65)	Weekly	18 (17)		
		Daily	40 (37)	Daily	7 (10)		
Smoking status	Pearson’s Chi^2^	Never	145 (142)	Never	34 (37)	1.19	0.55
		Former	12 (13)	Former	9 (7)		
		Current	25 (27)	Current	4 (3)		
No. of fillings	Pearson’s Chi^2^	None	44 (48)	None	16 (12)	2.93	0.40
		<5	84 (80)	<5	16 (21)		
		5–10	39 (40)	5–10	12 (11)		
		>10	13 (13)	>10	3 (3)		

## Data Availability

The authors will make data available upon reasonable request.

## Supplementary Materials

The following are available online at https://www.mdpi.com/article/10.3390/medicina58060715/s1, Figure S1: Information for participants and questionnaire.
